# Laying hen mortality in different indoor housing systems: a meta-analysis of data from commercial farms in 16 countries

**DOI:** 10.1038/s41598-021-81868-3

**Published:** 2021-02-04

**Authors:** Cynthia Schuck-Paim, Elsa Negro-Calduch, Wladimir J. Alonso

**Affiliations:** 1 Cartagena, Spain; 2 Epidemiology Research Group EPIDOT (Vice-Leader); Public Health Department, Federal University of Santa Catarina (UFSC), Santa Catarina, Brazil

**Keywords:** Animal physiology, Animal behaviour

## Abstract

Societal concern with the welfare of egg laying hens housed in conventional cages is fostering a transition towards cage-free systems in many countries. However, although cage-free facilities enable hens to move freely and express natural behaviours, concerns have also been raised over the possibility that cage-free flocks experience higher mortality, potentially compromising some aspects of their welfare. To investigate this possibility, we conducted a large meta-analysis of laying hen mortality in conventional cages, furnished cages and cage-free aviaries using data from 6040 commercial flocks and 176 million hens from 16 countries. We show that except for conventional cages, mortality gradually drops as experience with each system builds up: since 2000, each year of experience with cage-free aviaries was associated with a 0.35–0.65% average drop in cumulative mortality, with no differences in mortality between caged and cage-free systems in more recent years. As management knowledge evolves and genetics are optimized, new producers transitioning to cage-free housing may experience even faster rates of decline. Our results speak against the notion that mortality is inherently higher in cage-free production and illustrate the importance of considering the degree of maturity of production systems in any investigations of farm animal health, behaviour and welfare.

## Introduction

**Figure 1:**
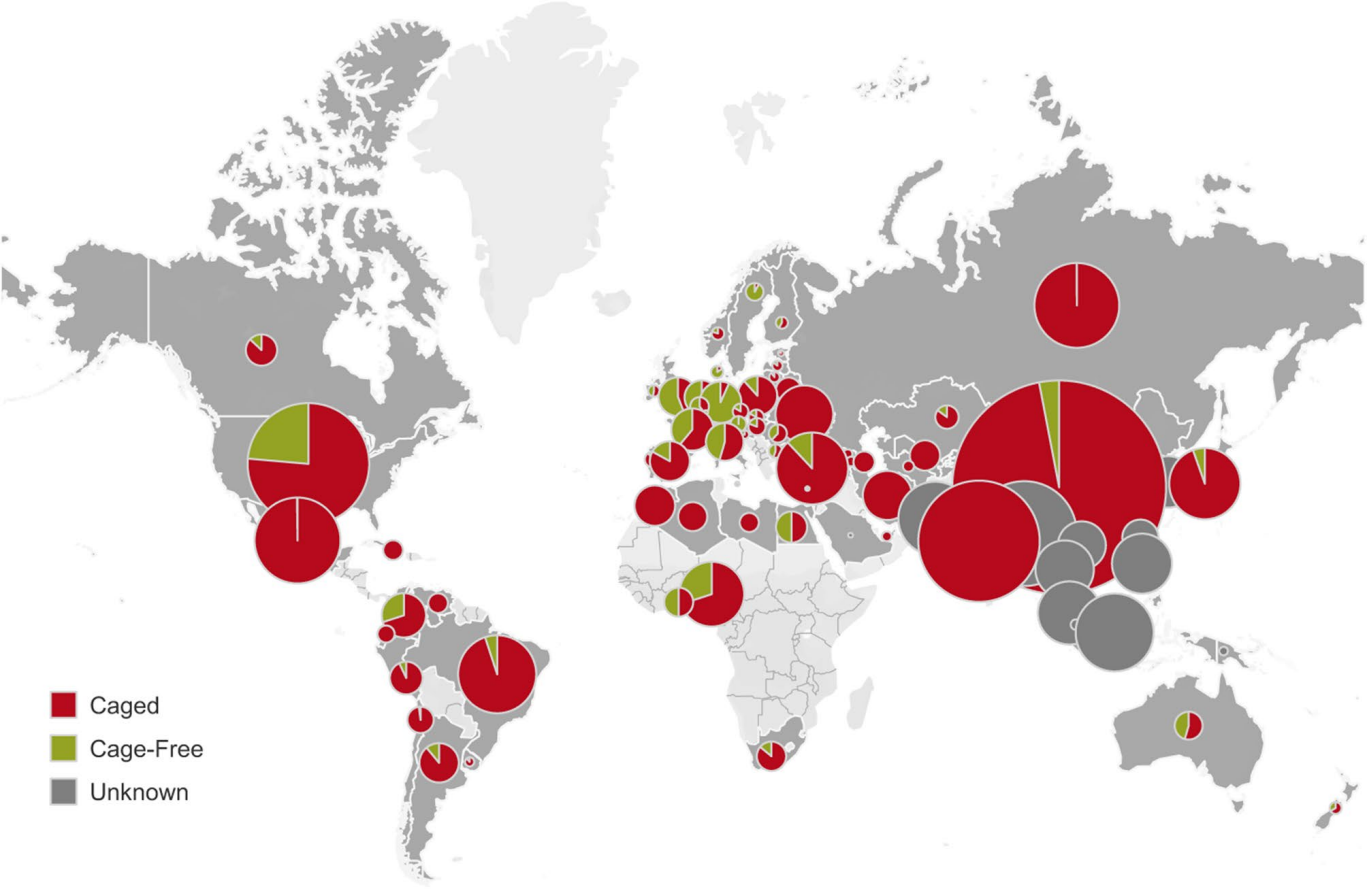
Global distribution of laying hens by housing system. Distribution of laying hens in caged (red: conventional; furnished) and cage-free (green: barns, aviaries, free-range) systems in 78 countries (dark grey) for which hen inventories are available. The size of circles is proportional to the hen population in the country. Dark grey circles: countries for which the hen population is known, but the proportion of hens in each housing system is unknown. An interactive version of this figure is available at http://hen-welfare.org/map, with further information available (by clicking on the pie charts) on data sources, proportion of hens in each system and layer populations. The map and graphic components were developed with the software Tableau Public, version 2020.3 (http://public.tableau.com).

Societal concern over the welfare of animals used in food production is on the rise, particularly with regard to the most intensive forms of animal farming, such as those used in the production of eggs. Currently, there are nearly the same number of egg laying hens alive (7.5 billion) as there are people on the planet^1^. The overwhelming majority (Fig. 1) are still kept in conventional cages (also known as battery cages), a form of extreme confinement that has been criticized for preventing individuals from moving freely, opening their wings and expressing natural behaviours they have a strong drive to perform, such as nesting, foraging, perching and dust bathing^2–4^.

Over the last 2 decades, public attention to the welfare of laying hens is fostering a gradual transition away from conventional cage systems, with an increase in the proportion of furnished cages (which provide hens with some additional space, a nest, perch and a litter substrate) and cage-free systems in some countries, initially in the European Union (EU Directive 1999/74/EC) and subsequently beyond Europe. For example, as of March 2020, nearly 24% of all hens in the United States were raised in cage-free systems, up from 12% in 2016 and 4% in 2010^5^.

Although cage-free facilities enable hens to move and express natural behaviours, concerns have also been raised over observations of higher mortality rates in these systems^6–9^. Flock mortality rates have been considered by some experts as one of the most important indicators of health for laying hens^10^, as higher death rates would be suggestive of poorer health. While a solid understanding of the causes of death in different housing systems is required to substantiate such a claim, should it be valid, it would imply that the health and welfare of egg-laying hens could be partially compromised following transition to cage-free systems.

However, whether mortality rates are higher in cage-free systems is not yet clear. Information on mortality is not systematically collated across the industry, and the very few reviews that have been conducted on the topic^7,11,12^ have shown inconsistent results. Where differences in mortality across housing systems were found^7^, they became nonsignificant when the confounding effect of beak trimming status was controlled (although beak trimming is a painful procedure with important negative effects on hen welfare^13,14^, its impact on reducing mortality due to injurious pecking is well known^15^).

A critical factor in the comparison of farm animal health and welfare across production systems is the potential difference in their degree of technological maturity. It is reasonable to expect that newly adopted systems will initially experience higher mortality due to lack of experience^16^, including the need to properly adapt diets, lighting schemes, rearing and management practices, and the design of the housing structure itself. Additionally, existing breeds might still be poorly adapted to cage-free systems, given decades of selection for caged environments. Appropriate comparisons would require managers, stock personnel and breeders having similar levels of experience with the systems, yet in most comparisons of mortality this premise was either not fulfilled^6,17^ or information on the degree of experience with a system was not available. To determine if laying hen mortality is inherently higher in a given production system, it is paramount to consider whether and how it changes as producers acquire experience with it. However, we are not aware of previous research on this topic.

We conducted a review and meta-analysis of existing evidence on laying hen mortality in commercial flocks raised in cage and indoor cage-free systems. To this end, we use data from 16 countries, 6040 flocks and over 176 million hens, making it the largest quantitative synthesis of commercial data on laying hen mortality to date. We focus on conventional cages, furnished cages and indoor aviary systems (single-tier and multi-tier), considered by the industry as the preferred cage-free alternative for large scale egg production. Next, we investigate the presence of temporal trends in laying hen mortality during the period from 2000 through 2020 in each of the housing systems studied.

## Results

### Search results and characteristics of the data sources

**Figure 2:**
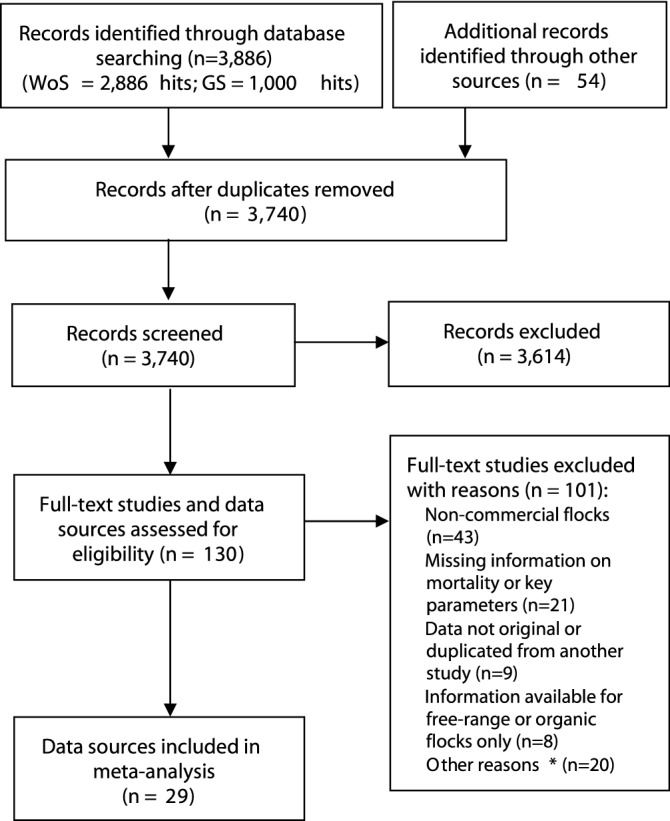
Flow diagram of selection process. Screening was conducted with the software JabRef version 5.0 (https://www.jabref.org). *A list with the reason for exclusion for each of the full-text papers screened is provided in the Supplementary Table S1.

A flowchart of the process of identification and selection of data sources is summarized in Fig. 2. Briefly, 3740 records of potentially eligible data sources were screened. Of these, 3614 were excluded for not meeting the pre-established inclusion criteria in the title and abstract screening stage, and 130 full-text articles and data sources were assessed for eligibility. At the full-text screening stage, 101 articles were excluded for various reasons, detailed in Fig. 2.

Overall, information from 29 data sources was included in the review, of which four datasets (Supplementary Table S2: FRA_16, NOR_18, URY_16, USA_13) correspond to survey data on layer mortality obtained from official sources or directly from authors. The data set covers 6040 flocks, with ~ 176 million hens observed from 2000 to 2019 in 16 countries. Of these, 4407 flocks were caged (3066 and 1341 in conventional and furnished cages, respectively), and 1633 flocks were in indoor cage-free systems: 412 and 290 in multi-tier and single-tier aviaries, respectively, and 931 flocks for which the type of aviary (single- or multi-tier) was not specified. The detailed description of the data sources included in the meta-analyses is summarized in Supplementary Table S2 and the supplementary data file (https://osf.io/r5f6c). Breed information was available for 14% of the flocks, with mortality figures often reported aggregated for multiple breeds. Of the flocks for which information on beak trimming status was available (3068), hens were mostly beak trimmed (84%), with approximately half of the flocks with intact beaks being from Norway, where beak trimming was banned in 1974.

### Mortality by housing system

**Figure 3:**
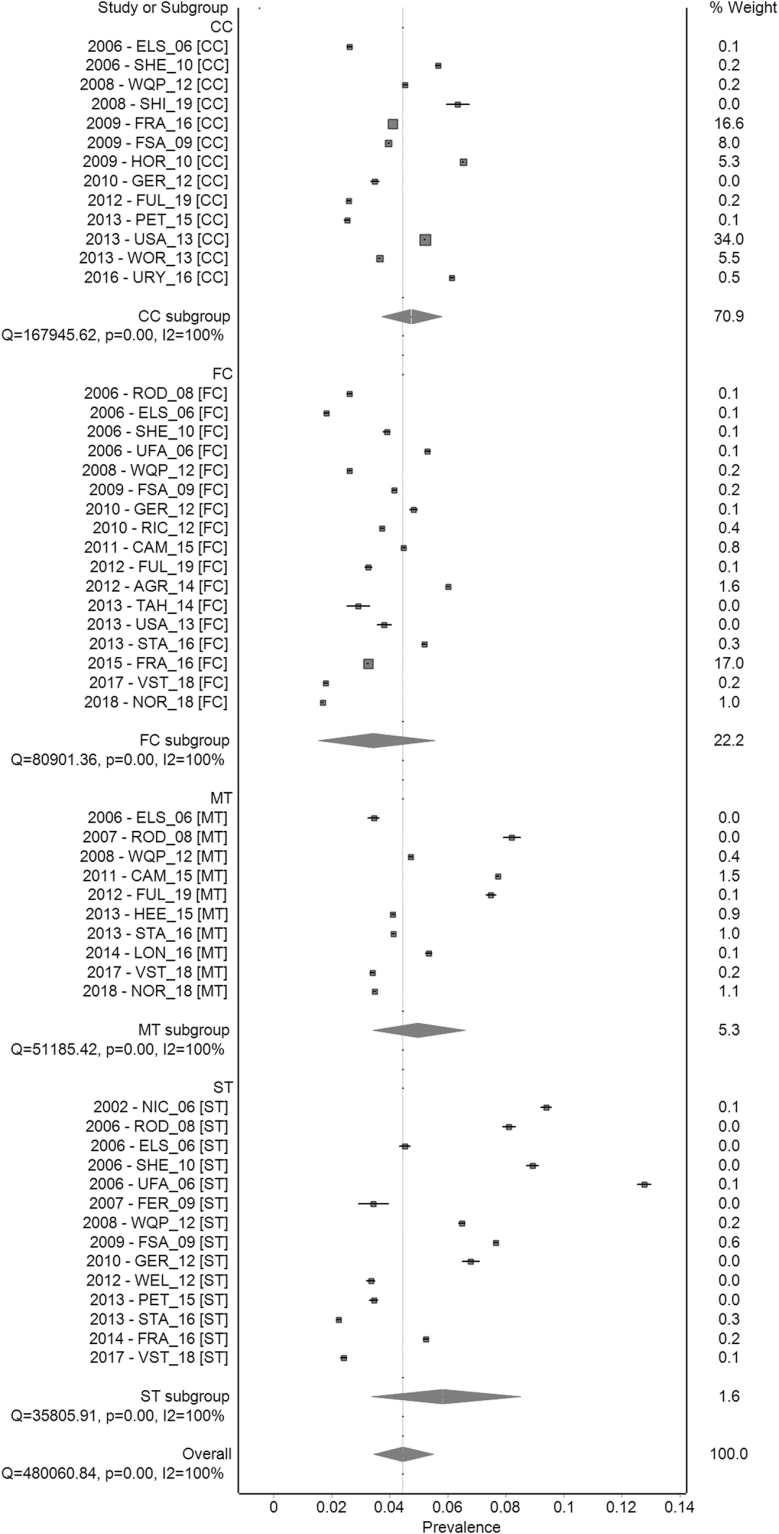
Forest plot of mean cumulative mortality of commercial layer flocks (2000–2020). Cumulative mortality at 60 weeks in different commercially managed indoor systems: conventional cages (CC), furnished cages (FC), single-tier (ST) and multi-tier (MT) aviaries, ordered by the mid-year of data collection. Each square represents individual estimates from each data source. Square sizes represent the weight derived from the meta-analysis (see Methods). Pooled estimates (weighted averages) are represented by diamonds.

Figure 3 shows cumulative mortality at 60 weeks for the cage and cage-free housing systems, ordered by the mid-year of data collection. Wide variations in mortality were evident in each housing system, with a noticeable trend towards lower mortality rates in more recent years in cage-free aviaries. Accordingly, substantial levels of heterogeneity within each system were observed (large Cochran’s Q-values with *p* < 0.01 and *I*^2^ > 99%), making it inappropriate to consider the pooled results.

**Figure 4:**
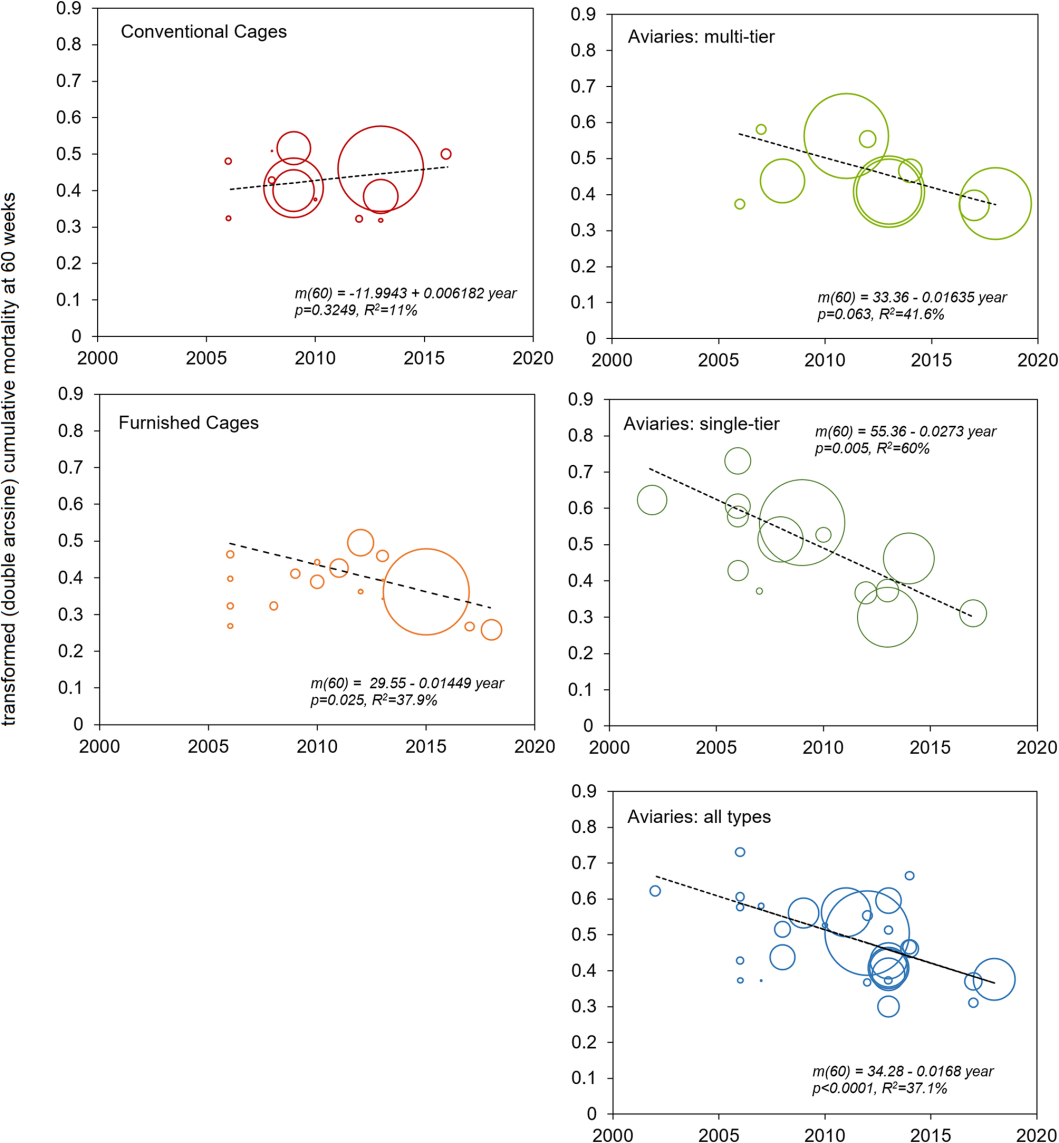
Meta-regression of cumulative layer mortality as a function of data collection year. Cumulative layer mortality at 60 weeks (m60, double arcsine transformed) in different commercially managed indoor housing systems. Regression equations and statistical results are shown in each corresponding graph. Each circle corresponds to one data source, with circle sizes representing the weights derived from the meta-analysis (see Methods).

The relationship between cumulative mortality and mid-year of data collection was thus explored in a meta-regression model (IVhet; see methods) using the full data set. Except for conventional cages, mortality levels were found to be progressively lower over the years for all housing systems (Fig. 4). A sensitivity analysis performed with the subset of the data (Table 1) for which variance in mortality was described by the data source similarly showed significant negative associations between mortality and year of data collection for all cage-free systems. Similar results were also found by conducting the meta-regression with the random effects model (Table 1). In an additional sensitivity analysis where we excluded those data sources for which we could not confirm that all flocks were kept exclusively indoors (multi-tier: HEE_15, NOR_18, VST_18; single-tier: VST_18; undefined aviary: USA_13), a similar pattern was observed, with significantly lower mortality in more recent years in single-tier aviaries and furnished cages (Supplementary Fig. S1). In all cases, the linear model was the best fitting regression model.

**Table 1:** Sensitivity Analyses. Meta-regression analyses of cumulative laying hen mortality in different commercially managed indoor housing systems as a function of mid-year of data collection using different meta-analytical models and subsets of the data.

Analysis	Data sources	Housing	N (sources)	Beta	SE	*p *value
(1) Subset of data: variance provided by data sources	Excluded (lack of information on variance): ELS_06; FER_09; GER_12; FUL_19; NOR_18; AGR_14; TAH_14	CC	9	− .0305	.0569	.609
		FC	11	− .0095	.021	.743
		ST	11	− .0878	.0224	.004
		MT	7	− .0909	.0331	.041
		AV	23	− .0767	.0145	.000
(2) Results from the random effects model	All data sources in Table S2	CC	13	− .0009	.0081	.905
		FC	17	− .0044	.0053	.4184
		ST	14	− .0225	.0049	.0006
		MT	10	− .0079	.0072	.3039
		AV	30	− .0132	.0044	.0060

Source codes are described in the Supplementary Table S2.

*CC* conventional cages, *FC* furnished cages, *ST* single-tier aviaries, *MT* multi-tier aviaries, *AV* indoor aviary (all types).

The association between cumulative mortality and additional pre-established risk factors for mortality (beak trimming status^15^, feather colour^18,19^ and flock size^7^) was also explored in uni- and multivariate analyses, shown in Table 2. Beak trimming status, feather colour and flock size were not associated with mortality in univariate analyses (i.e., not controlled for the year of data collection or the housing system). The effects of both year and housing were, in turn, highly significantly. Thus, we explored the effects of the explanatory variables in a multivariate model, testing whether the effect of housing on mortality depended on the year of data collection, as indicated in Fig. 4. As expected, a significant interaction between these two explanatory variables was observed, thus we conducted separate multivariate analysis for each housing system (Table 2). Year of data collection remained significantly associated with cumulative mortality in single-tier and all aviaries combined (the effect of year ceased to be significant in multi-tier aviaries, possibly due to the very few error degrees of freedom remaining in this case).

**Table 2:** Univariate and multivariate analyses on the effect of beak trimming (BT) status, feather colour, flock size, mid-year of data collection (year) and (indoor) housing system on cumulative layer mortality at 60 weeks (double arcsine transformed).

	Independent variable	F-value	df	*p *value
*Univariate analysis*				
	BT status (I,T,U)	0.57	(2,57)	0.571
	BT status (I,T) [excludes sources where BT = U]	0.00	(1,38)	0.975
	Feather colour (B,W,U)	0.28	(2,57)	0.759
	Feather colour (B,W) [excludes sources where FC = U]	0.44	(1,22)	0.515
	Flock size (log)	0.96	(1,58)	0.332
	Year	6.15	(1,58)	0.016
	housing (CC, FC, MT, ST, AV) **[FC < MT]	3.98	(4,55)	0.007
*Multivariate analysis*				
All housing systems	BT status (I,T,U)	1.83	(2,45)	0.172
	Feather colour (B,W,U)	0.13	(2,45)	0.878
	Flock size (log)	0.30	(1,45)	0.585
	Year	1.15	(1,45)	0.290
	Housing (CC, FC, MT, ST, AV) **[FC < MT]	2.68	(4,45)	0.044
	Year * housing	2.67	(4,45)	0.044
(1) Conventional	BT status (I,T,U)	0.42	(2,6)	0.674
Cage flocks	Feather colour (B,W,U)	1.36	(2,6)	0.326
	Flock size (log)	0.42	(1,6)	0.539
	Year	0.24	(1,6)	0.639
(2) Furnished	BT status (I,T,U) **[U > I;T]	4.68	(2,9)	0.040
Cage flocks	Feather colour (B,W,U)	0.50	(2,9)	0.621
	Flock size (log)	0.13	(1,9)	0.729
	Year	0.17	(1,9)	0.693
(3) Multi-tier	BT status (I,T,U)	0.01	(2,3)	0.995
Aviary flocks	Feather colour (B,W,U)	0.18	(2,3)	0.847
	Flock size (log)	0.67	(1,3)	0.474
	Year	0.01	(1,3)	0.995
(4) Single-tier	BT status (I,T,U)	3.69	(2,8)	0.073
Aviary flocks	Feather colour (B,W,U)	2.14	(1,8)	0.182
	Flock size (log)	0.54	(1,8)	0.483
	Year	16.67	(1,8)	0.004
(5) All aviary flocks	BT status (I,T,U)	1.00	(2,23)	0.384
(ST, MT, AV)	Feather colour (B,W,U)	0.11	(2,23)	0.893
	Flock size (log)	0.21	(1,23)	0.653
	Year	8.68	(1,23)	0.007

*I* intact beaks, *T* trimmed beaks, *W* white hybrids, *B* brown hybrids, *U* unknown beak status or feather colour, *CC* conventional cages, *FC* furnished cages, *MT* multi-tier aviary flocks, *ST* single-tier aviary flocks, *AV* unspecified aviary type.

**Tukey pairwise comparisons for categorical factors.

**Figure 5:**
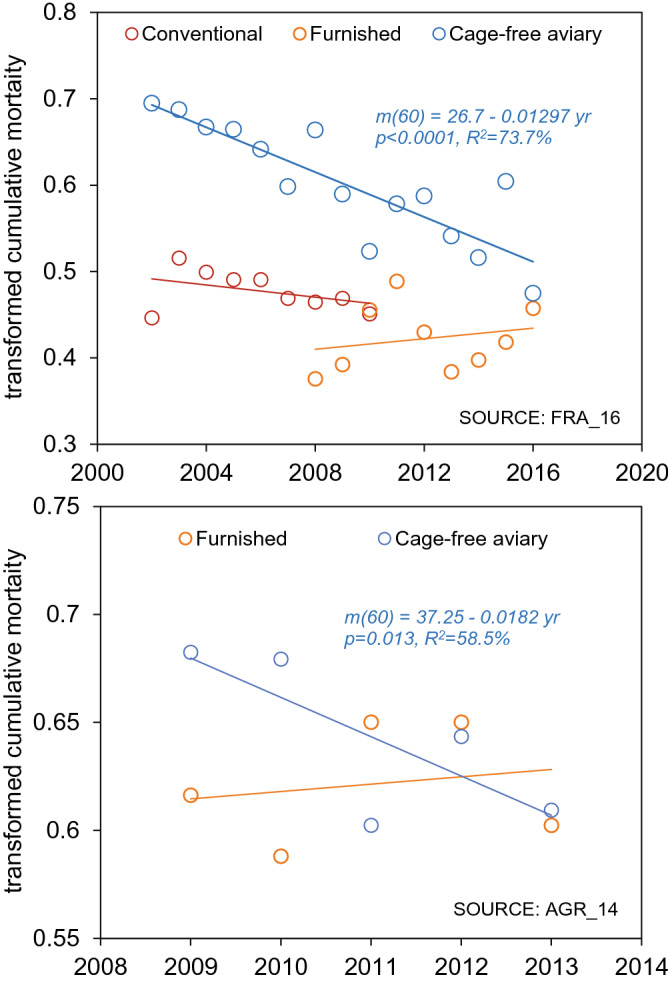
Time series of cumulative layer mortality for two independent data sources. Regression of cumulative mortality rate (m60, double arcsine transformed) as a function of year of data collection for commercial flocks raised in conventional cages, furnished cages and a non-cage system (single-tier aviaries) in France ( source code FRA_16) and the Netherlands (AGR_14). Regression equations are shown where the effect of year is significant.

To further investigate the relationship between mortality and time in homogeneous time series data from single, independent, data sources (hence unconfounded by regional differences in production practices or other factors that may differ across data sources), we analysed the surveys from (1) the Technical Institute for Poultry in France (ITAVI), from which data on cumulative hen mortality over a period of sixteen years (2002–2016) were available (data source FRA_16), and (2) from Agrovision (AGR_14), as reported by Leenstra and collaborators^20^, for which data for commercial flocks in the Netherlands was reported for five consecutive years (2009–2013). For both France and the Netherlands, no temporal trends in mortality were detected for caged systems, but there was a highly significant drop in mortality over this period in the cage-free aviaries, with year of data collection accounting for 73% and 58% of the variability in mortality reported in the two countries, respectively (Fig. 5).

**Figure 6:**
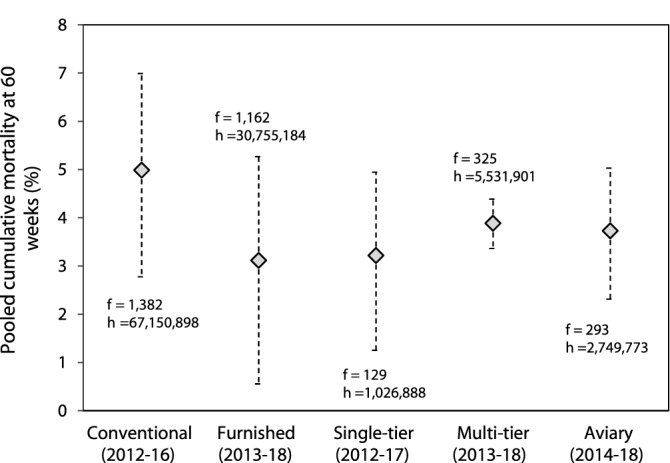
Pooled estimates of cumulative layer mortality in recent years. Pooled estimates of cumulative layer mortality at 60 weeks (95% CI) using the five most recent data sources for each commercially managed indoor housing system (conventional cages, furnished cages, single-tier aviaries, multi-tier aviaries and all types of aviaries). Years of data collection are shown below each housing system (*f*: number of flocks; *h* = number of hens).

Table 3 describes the mean percent reduction in mortality per year based both on the meta-regression results and the regression analysis of the time series data from individual data sources. The results indicate an average decline in mortality of approximately 0.35–0.65% per year in the cage-free aviaries. These results indicate that it is inappropriate to pool evolving mortality data from far apart years. Thus, for each housing system, we restricted the meta-analyses to the five most recent data sources available, providing a window to reduce temporal variation while avoiding significant error by including outdated figures. Pooled mortality estimates are shown in Fig. 6 for each housing system—no significant differences among systems were observed (*F*_3,16_ = 0.77, *p* = 0.525).

**Table 3:** Mean percent reduction in laying hen mortality in different indoor housing systems at 60 weeks per year (95% CI) as determined from meta-regressions (Fig. 4) and for independent data sources (Fig. 5: FRA_16; AGR_14). Coefficients were back-transformed to natural proportions.

Mean percent reduction in mortality per year (95% CI)			
	Meta-Regression (Fig. 4)	FRA_16 (ITAVI) (Fig. 5)	AGR_14 (Fig. 5)
Conventional cages	*p* = NS	*p* = NS	No data
Furnished cages	0.28% (0.05–0.55)	*p* = NS	*p* = NS
Single-tier aviaries	0.65% (0.29–0.99)	No data	No data
Multi-tier aviaries	0.37% (0.03–0.79)	No data	No data
All aviaries	0.46% (0.27–0.67)	0.37% (0.25–0.47)	0.55% (0.35–0.75)

## Discussion

Our results provide analytical confirmation for the expectation that mortality should gradually drop following the transition to new housing systems and speak against the notion that mortality is inherently higher in cage-free production. Using multiple data sources from several countries, we have shown that while flock mortality in conventional cages seems to have reached a plateau, each new year of experience with an indoor cage-free system has been associated with a 0.4–0.6% average drop in mortality (or 4–6% over a decade).

The observation of a robust and strong association between layer mortality and the degree of maturity of the production system was consistently replicated with different meta-analytic models and in different sensitivity analyses, and further validated with two independent datasets (Fig. 5), where a strong and significant decline of mortality in cage-free aviaries, within the same country, was observed over time. These results also find support in various prior observations. For example, in a survey of Flemish farmers, mortality at 65 weeks was significantly higher in newly introduced furnished cages (5.8%) than in single-tier aviaries (2.5%), when experience with furnished cages was of 4 years compared to ten years for aviaries^21^. Leenstra et al^20^ also report data showing a decrease in mortality among hens housed in aviaries and organically in Switzerland, France and the Netherlands, with mortality dropping to levels comparable to other traditional systems over the years. The same pattern was observed in Norway, where mortality at 60 weeks dropped from 10.3% in 2003 to 3.3% in 2010 in free-range aviaries^22^ (online supplement: hen-welfare.org/time-series). Similarly, higher mortality rates, above 10%, were typical of conventional cages in the past^23,24^.

For decades, industrial cage systems have been adjusted to reduce losses caused by the death of productive individuals, and it is natural that newly adopted housing systems follow the same path. Not only is there a direct financial incentive to reduce mortality, but risk factors for mortality in cage-free systems (such as new pathogen challenges and exposure to injurious pecking) can be managed if producers can acquire the experience needed to run cage-free systems successfully. For example, fifteen years after battery cages were banned in Switzerland, infectious diseases frequently encountered in other countries were mostly absent in Swiss flocks due to the development of proper hygiene protocols and vaccination programs^25^. Similarly, the adoption of preventive strategies against feather pecking has been shown to significantly reduce levels of injurious pecking in aviaries^26,27^. As knowledge on practices evolves, housing design improves and genetics are progressively optimized for loose housing facilities^28^, new producers transitioning to these systems may experience even faster rates of decline in mortality than those reported here. Conversely, less steep reduction rates may be observed in those countries and regions (other than western Europe and North America, where most of the data reported here originates) that lack the financial and human resources needed for the dissemination of knowledge, proper training and the implementation of best practices.

When compared over a recent period, differences in mortality among caged and cage-free systems were no longer evident, with average figures ranging from 3 to 5% at 60 weeks (Fig. 6). These figures are, however, limited by the low availability of data to control for putative differences in the prevalence of brown and white hybrids among the housing systems, as for most data sources there was either no information on the breeds investigated or mortality figures were averaged for multiple breeds. Because brown hybrids may experience higher mortality^18,19^, differences in mortality across systems might be affected by the extent to which their prevalence differed across the systems. Additionally, the analysis of a more recent period does not ensure that the maturity of the systems in this recent data set was comparable. Considering that cage-free facilities are likely to have more room for progress, and that brown-feathered genotypes are more common in cage-free systems, it is possible that mortality in cage-free systems drops further when these two factors are taken into account. Nevertheless, it is not possible to rule out the possibility that mortality is underreported in cage-free houses, as it is likely more difficult to identify and capture dead birds in these facilities^16^.

One could also argue that cage-free hens experience higher mortality at ages older than 60 weeks, as suggested by observations that cage-free flocks tend to be depopulated earlier^21^. However, evidence indicating a higher mortality at older ages as a key driver of earlier depopulation is not strong. For example, in the United States, cage-free flocks are depopulated when mortality reaches on average 6.4%, compared to 10.5% for flocks raised in conventional cages^29^. The greater facility with which caged flocks are molted (a practice still widely adopted outside Europe, that itself raises important welfare concerns) gives producers a reason to extend their lives, with 60–80% of commercial caged layers being molted in that country (on average at 65–70 weeks)^29–31^, compared to less than 15% in cage-free production^29^.

Naturally, the meta-analytic findings reported here are limited by the quantity, quality, and representativeness of the data sources included. For example, not all regions were represented in the eligible data sources identified, with the analysis mostly limited to countries in Europe and North America. Particularly, no data was identified for many of the top egg producing countries (e.g. China, India, Mexico, Brazil). The extent to which the findings can be generalized to other parts of the world thus remains to be determined. Additionally, important sources of heterogeneity (such as beak trimming and breed) were not reported by many data sources, limiting the extent to which their effects could be properly analysed. Also, precise information on sample sizes, variances or the raw (disaggregated) data (from individual flocks) enabling their calculation was missing in many cases. Although sensitivity analyses restricted to a subset of data for which this information was available confirmed our findings, these observations highlight the benefits that could be accrued by more comprehensive reporting. Incentives for the publication of (already collected) anonymized data from commercial farms (e.g. by breeding companies) should greatly help establish the evolution of the industry and provide insights into effective strategies to improve layer welfare.

Despite our observation of comparable mortality in different housing systems, a solid understanding of variations in the causes of death across systems is also essential if meaningful associations between mortality and health or welfare are to be suggested. Even if a system were associated with longer survival or lower mortality, it would not necessarily indicate better health or welfare. For example, health conditions producing weakness, lethargy, or reduced movement are likely prolonged over longer periods in cage systems, as no displacement is needed for sick or critically ill individuals to keep eating and drinking, and energy expenditure is lower. This allows for a lower mortality, but a higher prevalence of morbid individuals. Additionally, mortality fails to capture the impact of nonfatal outcomes of disease, injury and deprivations on welfare. Put simply, what makes animals suffer is not necessarily what kills them. Finally, the loss of welfare imposed by fatal outcomes also depends on their nature. For example, deaths due to painful reproductive disorders and infectious diseases are possibly associated with individuals in states of poor welfare in the flock. Conversely, in cases such as predation and accidents, animals in good health can become fatalities. We can illustrate this phenomenon with a well understood example from humans: children who never play outdoors are less prone to injuries and fatal accidents, but it does not mean that they are healthier or happier. However, systematic research on the causes of normal hen mortality in different commercial systems, based on random samples of dead birds, is scant^6,9,32,33^ and likely confounded by the effects discussed here. Future research should address this gap.

Our findings also have important implications in other areas of animal welfare science. Specifically, the demonstration that the degree of maturity and experience with a system acts as a major confounder in analyses of mortality suggests that other comparisons of health, behaviour and welfare across systems may be similarly affected by this factor. The interpretation of the literature in these other areas, including results obtained in experimental settings, should take this possibility into account.

## Methods

### Search strategy

We identified potentially eligible data sources using several methods. First, we searched Web of Science (Core collection) for relevant studies from 2000 until 2020 using the following topic search strategy: (“laying hen” OR layer) AND (aviar* OR cage* OR housing OR barn) AND (mortality OR death OR performance OR survival OR productivity OR welfare) (Supplementary Table S3). Given the nature of the research question and data (commercial data on cumulative layer mortality), a comprehensive search of the grey literature (articles not formally published by commercial academic publishers) and data from surveys (e.g. governmental surveys of commercial farms) was also required. To this end, we complemented the search with Google Scholar (GS) to increase coverage given its high sensitivity, using the same search terms and range of dates (GS restricts the results to the first 1000 records retrieved^34^). In addition, bibliographies of key articles and reviews were hand searched. Articles in English, Spanish, Portuguese and French were considered in this screening process. We also searched the websites of the Agriculture and Statistics departments of English-, Spanish-, Portuguese- and French-speaking countries for unpublished data sources and surveys. The Meta-analysis Of Observational Studies in Epidemiology (MOOSE) statement^35^ was used to guide reporting.

### Selection criteria

Identified data sources, whether articles or survey data, had to meet all of the following criteria to be included: (1) report cumulative mortality (from the year 2000 onwards) and the corresponding age of the flock, or data (e.g., weekly, monthly mortality) enabling the calculation of cumulative mortality at specific ages; (2) report data from commercial flocks (defined as commercial facilities managed by stock personnel, hence excluding research stations and facilities operated exclusively by researchers) with at least 1000 hens; (3) specify (or enable the estimation of) the year of data collection; (4) for data sources reporting aggregate mortality for multiple flocks, report sample size (number of hens and flocks) or data enabling its estimation and (5) report mortality disaggregated by housing system, including at least one of the following housing systems, defined as in Weeks et al^7^: conventional cages, furnished (enriched) cages, multi-tier aviaries (a house where birds have access to several tiers, litter at the ground level, and perches and nests on one or more of the other tiers); and single-tier aviaries (or barns), representing a house with litter at the ground level and other resources on a raised area.

### Study selection, data extraction and processing

An initial screening stage was conducted by reading the title and summary of each of the potentially eligible data sources retrieved with the software JabRef version 5.0^36^. Studies that clearly did not meet the inclusion criteria were eliminated at this stage. In other cases, or if not enough information was provided on the summary, the data source was passed on to the next round. Along with cross-references, a total of 130 full papers and data sources were analysed, and data from eligible studies extracted by means of a standardized protocol. In an effort to include more studies for which critical information was missing, we contacted the authors for clarification. All screening and data extraction stages were conducted independently by two co-authors (CSP and ENC). Where disagreements existed, the co-authors reviewed the articles once more and discussed the discrepancy until a consensus was reached. Discrepancies were few and predominantly the result of missing information on articles, resolved in most cases by contacting the authors of the articles for further clarification. Adjudication by a third reviewer was planned but not necessary.

For each data source, we identified one or more cohorts of animals, where a cohort is defined as the largest group of animals from a housing system for which the necessary data meeting the inclusion criteria were available. For each cohort, the following variables were extracted: data source information, country, housing system, year(s) of data collection, population characteristics (breed, beak trim status), sample size (number of hens, flocks, farms), flock size, cumulative mortality, variance (if summary measures of mortality were provided) and age (in weeks) for which cumulative mortality was reported. Other variables descriptive of housing conditions were also collected if available, including years of experience with the system, mean density (animals/m^2^), animals per cage, rearing system, presence or prevalence of molting and mean light intensity (lux). Where quantitative information was only available in charts, we used WebPlotDigitizer version 4.2 for data extraction^37^.

While most data sources reported only summary measures, namely mean mortality figures for multiple flocks, some reported mortality data for each individual flock. In such cases, we calculated the average and standard error of mortality for the flocks of each housing system (data from free-range or organic flocks were excluded), provided the data were restricted to a period of three or fewer years and the flocks did not mix laying hens with different beak statuses and feather colours. In the few cases where the same data source reported mortality for multiple years (e.g., AGR_14, FRA_16; Supplementary Table S2), we considered the last three years of data available. Where data sources provided data on mean mortality for multiple flocks and the range of mortality observed (minimum and maximum values), but no measure of variance in mortality across flocks, standard errors were calculated from the range using the method of Hozo and collaborators^38^.

### Age standardization

Because cumulative mortality increases with flock age, and different data sources report mortality at different ages, mortality figures were standardized, where appropriate, to reflect mortality at 60 weeks. The threshold of 60 weeks was chosen not only because it was the predominant reporting age, but also because mortality at a later age is likely to be confounded by differences among systems in the prevalence of induced molting, which is still a widespread practice in countries outside the United Kingdom and European Union and usually occurs between 65 and 70 weeks^29–31^.

Where mortality was reported at different (predominantly later) ages, age adjustment was conducted by determining the average weekly mortality for the specific flocks from the laying onset until the age for which mortality was reported, so that weekly mortality = reported mortality/(reporting week − laying onset week). Next, a linear transformation was employed to determine mortality at 60 weeks by multiplying the average weekly mortality by the number of laying weeks (60—laying onset week). A linear model was chosen for its good fit with the mortality data, particularly at later ages, shown in studies that followed mortality over time^17,39–41^. This procedure provides a better approximation than that obtained with the use of regression coefficients (relating mortality and flock age across multiple data sources), as the proportion of variance that remains unexplained by such a regression is high given the many differences in risk factors for mortality (e.g., breed, management practices, beak trim status, flock size, lighting conditions) across sources. Conversely, age adjustments based on a population’s own weekly mortality are not confounded by these effects.

### Data analysis

The primary outcome was cumulative layer mortality at 60 weeks. To obtain a quantitative synthesis of the mortality estimates, age-standardized mortality was pooled through a meta-analysis. In meta-analysis, a weighted average is obtained to summarize the magnitude of the variable measured through a set of primary data sources. The weight assigned to each estimate is often set proportional to the inverse of its variance, so larger studies with more precise estimates have more weight and smaller, noisier studies have less weight. It is well established, however, that the calculated precision of meta-analytic findings using the fixed effects model is overstated in the presence of heterogeneity^42,43^ (as expected in the present case, given the multiple factors affecting layer mortality). Because error estimation and coverage also remain inadequate with currently utilized random effects (RE) models^44–47^, we chose to use as our main analytic approach a meta-analytic model that solves this issue, the IVhet model^46^. The IVhet model has been shown to be an improved alternative to the RE model, giving larger weights to studies with larger samples and providing more conservative confidence intervals^46^, effectively solving the problem of error estimation as demonstrated in simulation studies^48^. The results of the RE model are reported for comparison.

Because some data sources did not report variance in mortality across the flocks studied (nor provided the raw data enabling its calculation), we conducted a typical meta-analysis of prevalence (mortality, expressed in proportion), having the total number of hens (*N*) of each data source as its population size, and variance calculated using the binomial equation *p*(1 − p)/N^49^. Where the total number of hens was not described, it was estimated as the product between the number of flocks and the mean flock size for the housing system (as given by the data source or, if unavailable, as the average for the housing system in the country or region). Mortality was transformed using the double arcsine square root transformation to stabilize the variance across data sources^49^. Heterogeneity was evaluated by Cochrane *Q* and *I*^2^ statistical methods.

Meta-regressions were conducted to investigate potential trends in mortality over time. Robust standard errors were used to allow for correct error estimation. Additionally, where data sources allowed, we examined whether mortality was affected by the following moderator variables, defined a priori: beak trim status (BT), flock size and hybrid colour (brown, white feathered birds), where BT and hybrid colour were defined as categorical (fixed effects) variables, and flock size and year of data collection as continuous variables. Hybrid colour was used instead of breed since the size of the sample for each breed was insufficient to enable its inclusion as a factor in the analyses. Similarly, because most data sources provided aggregated information about multiple flocks, the analysis of factors that tend to be highly specific to individual flocks, such as the size of subunits (or pens) in multi-tier aviary systems, was not possible.

Where datasets included mortality data for multiple years, we also investigated temporal trends in cumulative mortality within each data source. To this end, we fitted a regression model having the (double arcsine square root) transformed mortality estimates as the dependent variable, and mid-year of data collection as the independent variable. For ease of interpretation, results were back-transformed to natural proportions^49^. Pooled analyses were conducted using MetaXL version 4.3 (EpiGear International, Queensland, Australia)^50^, and the meta-regression models were run using Stata SE version 14 (Stata Corp, College Station, TX).

## Supplementary information

Supplementary Information 1.

## Data Availability

Data is available at https://osf.io/r5f6c.
