# Supplementary material for: Laying hen mortality in different indoor housing systems: a meta-analysis of data from commercial farms in 16 countries
Source: Sci Rep. 2021 Feb 4;11:3052. doi: 10.1038/s41598-021-81868-3 (PMC7862694; doi:10.1038/s41598-021-81868-3)
Supplement: Supplementary file 1 — Supplementary Information 1. [file 41598_2021_81868_MOESM1_ESM.pdf]

Supplementary Material

Laying hen mortality in different housing systems: a meta-analysis of data from commercial farms in 16 countries

Authors: Cynthia Schuck-Paim; Elsa Negro-Calduch, Wladimir J. Alonso

Table S1. References excluded at the full-text screening stage.

First Author	Year	Title	Exclusion Reason
AgraCEAS	2004	Study on the socio-economic implications of the various systems to keep laying hens	No information on flock ages, or data enabling calculation of cumulative mortality at specific ages; some estimates do not seem to be empirically collected
Ahammed	2014	Comparison of aviary, barn and conventional cage raising of chickens on laying performance and egg quality	Non-commercial
Amin	2013	Behaviour of laying curve in Babcock-380 brown commercial	Lacks: Production system description.
Anderson	2007	Effects of alternative molting programs and population on layer performance: Results of the thirty-fifth North Carolina layer performance and management tests	Non-commercial
Appleby	2002	Development of furnished cages for laying hens	Non-commercial
Aral	2017	Economic comparison of unenriched and alternative cage systems used in laying hen husbandry - recent experience under Turkish commercial conditions	Non-commercial
Berg	2001	Health and welfare in organic poultry production.	Organic only
Bestman	2004	Health in organic laying hens – facts and fairy tales. Proc. of the 2nd SAFO Workshop, Witzenhausen, Germany, pp. 145–152.	Organic only
Blatchford	2016	The utilization of the Welfare Quality assessment for determining laying hen condition across three housing systems	Duplicate data with Fulton 2019
Blokhuis	2007	The LayWel project: welfare implications of changes in production systems for laying hens	Mixed data on commercial and small experimental trials. No information on hen age
Cabezas	2011	Analisis de la interacción genotipo x ambiente entre sistemas de alojamiento y razas de gallinas ponedoras para indicadores de bienestar y calidad de huevo	Non-commercial
Cabrelon	2015	Bem-estar animal: densidades de ocupação em gaiolas convencionais de produção de ovos e seus reflexos nos índices produtivos	Non-commercial
Cande	2016	Avaliação do desempenho produtivo de poedeiras comercias da linhagem Hy-Line Silver Brown na empresa Galovos Lda. no distrito de Boane, Maputo	Non-commercial
Comin	2019	Revealing the structure of the associations between housing system, facilities, management and welfare of commercial laying hens using Additive Bayesian Networks	Mortality data (by housing) is aggregated for conventional and organic flocks
Croxall	2007	The comparative welfare of laying hens in a wide range of egg production systems as assessed by criteria in Swedish animal welfare standards	Lacks: Mortality Information

DEFRA	2009	The influence of rearing environment on propensity for injurious pecking in laying hens.	Non-commercial
Demli	2018	Comparing the performance and egg quality of two strains of laying hens housed in furnished cages	Non-commercial
Demli	2016	Comparative performance and egg quality of laying hens in enriched cages and free-range systems	Non-commercial
Dickmen	2016	Egg production and welfare of laying hens kept in different housing systems	Non-commercial
Drake	2010	Influence of rearing and lay risk factors on propensity for feather damage in laying hens	Lacks: Mortality Information
EFSA	2005	Opinion of the Scientific Panel on Animal Health and Welfare on a request from the Commission related to the welfare aspects of various systems of keeping laying hens	Review. Data from before 2000
EFSA	2015	Scientific Opinion on welfare aspects of the use of perches for laying hens	Does not apply to research question
Eladl	2018	Prevalence of mites and their impact on laying hen (Gallus gallus domesticus) farm facilities in Egypt, with an analysis of deltamethrin residues in eggs and tissue	Non-commercial
Elson	2015	Poultry welfare in intensive and extensive production systems	Review
Englmaierova	2014	Effects of laying hens housing system on laying performance, egg quality characteristics, and egg microbial contamination	Lacks: Mortality Information
Estevez	2014	ISAE2014: proceedings of the 48th congress of the International Society for Applied Ethology, Vitoria-Gasteiz, Spain.	Lacks: Mortality Information
Fossum	2009	Causes of mortality in laying hens in different housing systems in 2001 to 2004	Convenience sample (not cumulative mortality)
Freire	2013	The welfare of laying hens in conventional cages and alternative systems: first steps towards a quantitative comparison	Meta-Analysis of other authors' data
Golden	2012	A comparative examination of rearing parameters and layer production performance for brown egg-type pullets grown for either free-range or cage production	Non-commercial
Gómez	2010	Evaluación del bienestar animal y comparación de los parámetros productivos en gallinas ponedoras de la línea Hy-Line Brown en tres modelos de producción …	Non-commercial
Guesdon	2004	Humeral quality and adrenal responsiveness in laying hens reared in standard and furnished cages	Non-commercial
Guesdon	2004	Laying performance and egg quality in hens kept in standard or furnished cages	Non-commercial
Guinebretiere	2013	Plumage condition, body weight, mortality, and zootechnical performances: The effects of linings and litter provision in furnished cages for laying hens	Non-commercial
Hagan	2013	The egg production performance of three layer strains kept under intensive system in the hot and humid tropics	Non-commercial
Hane	2000	Survey of laying hen husbandry in Switzerland	Data from before 2000
Heerkens	2013	Do keel bone deformations affect egg-production in end-of-lay laying hens housed in aviaries?	Duplicate data
Heerkens	2014	Effect of aviary housing characteristics on laying hen welfare and performance	Duplicate data
Hegelund	2006	Welfare and productivity of laying hens in commercial organic egg production systems in Denmark	Free-range/organic only
Henry	2002	Organic Poultry - Eggs. Maritime Certified	Lacks: Mortality information

Hetland	2004	Effect of including whole oats into pellets on performance and plumage condition in laying hens housed in conventional and furnished cages	Non-commercial
Huneau-Salaun	2011	Endotoxin concentration in poultry houses for laying hens kept in cages or in alternative housing systems	Non-commercial
Iannaccone	2013	Productive performance and egg quality of laying hens raised in conventional and furnished cages	Mortality seems to be mean monthly mortality, makes it difficult to derive accumulated mortality in the presence of induced molting procedure.
Itza-Ortiz	2016	Productive performance of white Leghorn hens based on the type of housing during hearing: floor versus cage.	Restricted period of observation, not possible to calculate age-specific mortality at 60 weeks
Jendral	2008	Bone Mineral Density and Breaking Strength of White Leghorns Housed in Conventional, Modified, and Commercially Available Colony Battery Cages	Lacks: Mortality Information
Karcher	2015	Impact of commercial housing systems and nutrient and energy intake on laying hen performance and egg quality parameters	Duplicate data with Fulton 2019
Krivankova	2020	Comparison of selected indices of internal environment and condition of laying hens kept in furnished cages and in aviaries	Lacks: Mortality Information
Landman	2015	The incidence and economic impact of the Escherichia coli peritonitis syndrome in Dutch poultry farming	The age for which mortality in reported not clear
Leenstra	2012	Performance of commercial laying hen genotypes on free range and organic farms in Switzerland, France and the Netherlands	Free-range/organic only
Leyendecker	2005	Keeping laying hens in furnished cages and an aviary housing system enhances their bone stability	Lacks: Mortality Information
Louton	2017	Evaluation of welfare parameters in laying hens on the basis of a Bavarian survey	Mortality data aggregated for all systems (aviaries, free-range, furnished cages). Aggregated data based on survey.
Méndez	2010	Aplicabilidad de un protocolo de medición de bienestar animal creado por el proyecto Welfare Quality® en gallinas de postura comercial en jaulas	Lacks: Mortality Information
Mielenz	2005	Mortality of laying hens housed in single and group cages	Article in German, mortality reported at 6 months (not end of lay), not clear if conditions are commercial
Morrissey	2016	Can Non-Beak Treated Hens be Kept in Commercial Furnished Cages? Exploring the Effects of Strain and Extra Environmental Enrichment on Behaviour, Feather Cover, and Mortality	Non-commercial
Mugnai	2009	Effect of rearing system and season on the performance and egg characteristics of Ancona laying hens	Non-commercial
National Chung-Hsing University Thesis	2015	Investigating the welfare of laying hens in the conventional cage and floor production systems by Welfare Quality®	Chinese, Experimental
Nicodemus	2012	Efecto de la densidad de gallinas por jaula y de la estirpe sobre la producción y la calidad del huevo	Very small sample, likely non-commercial
Oden	2002	Behaviour of laying hens in two types of aviary systems on 25 commercial farms in Sweden	Lacks: Mortality information
Oliveira	2014	Performance and quality of egg laying hens raised in furnished cages and controlled environment	Lacks: Mortality Information
Olivera	2019	Effects of litter floor access and inclusion of experienced hens in aviary housing on floor eggs, litter condition, air quality, and hen welfare	Non-commercial
Peralta	2016	Productive performance and behavior of laying hens in three housing systems under tropical conditions	Open systems exposed to weather conditions

Peralta	2016	Desempeño productivo y conductas etológicas de gallinas ponedoras en tres tipos de manejo en condiciones de trópico cálido	Non-commercial
Pereira	2010	Estimating mortality in laying hens as the environmental temperature increases	Lacks: Mortality Information
Philippe	2020	Comparison of egg production, quality and composition in three production systems for laying hens	Non-commercial
Pohle	2009	Furnished cage system and hen well-being: Comparative effects of furnished cages and battery cages on behavioral exhibitions in White Leghorn chickens	Lacks: Mortality Information
Popescu	2012	Battery Change Improves the Welfare of Laying Hens, Animal science and Biotechnologies 45, 250 (2012).	Not clear if the cage system is exposed to weather. Not clear if induced molting was practiced (as indicated by high mortality in cages at 78 weeks)
Rakonjac	2014	Laying hen rearing systems: a review of major production results and egg quality traits	Review
Regmi	2018	Effects of different litter substrates and induced molt on production performance and welfare quality parameters of white Leghorn hens housed in multi-tiered aviary …	Non-commercial
Rivera	2016	Evaluación de pico de postura y persistencia de la producción en gallinas ponedoras de las líneas Hy-line W-36 y Hy-Line CV-24de 78 semanas de edad	Lacks: Production system description. Non-commercial setting
Rodrigues	2011	Desempenho e qualidade de ovos de galinhas poedeiras de uma granja em região de clima tropical	Mortality reporting inconsistent (text and chart in Annex A). Conventional cage in open systems (exposed to weather)
Roll	2005	Bienestar animal y productividad en gallinas ponedoras comerciales alojadas en jaulas enriquecidas	Non-commercial
Roll	2008	Aspectos etológicos y productivos de gallinas ponedoras alojadas en jaulas convencionales o enriquecidas de fabricación española	Duplicate data with Roll 2008
Sherwin	2013	Prevalence of nematode infection and faecal egg counts in free-range laying hens: relations to housing and husbandry	Lacks: Mortality Information
Shimmura	2007	Behavior, physiology, performance and physical condition of layers in conventional and large furnished cages in a hot environment	Non-commercial
Shimmura	2010	Multi-factorial investigation of various housing systems for laying hens	Non-commercial
Shittu	2014	Predictors of death and production performance of layer chickens in opened and sealed pens in a tropical savanna environment	Production in conventional cages, open system (mortality estimates excludes culling of aggressive, sick and poorly producing birds)
Silva	2013	Performance of laying hens and economic viability of different cimatization systems	Only mean daily mortalities (aggregated over the entire period) are provided. Two of the systems seem to be exposed to weather conditions.
Singh	2009	Production performance and egg quality of four strains of laying hens kept in conventional cages and floor pens	Non-commercial
Sirovnik	2018	Feeding from perches in an aviary system reduces aggression and mortality in laying hens	Non-commercial
Sirovnik	2018	Feeder space affects access to the feeder, aggression, and feed conversion in laying hens in an aviary system. Appl. Anim. Behav. Sci. 198:75–82.	Non-commercial
Sosnówka	2010	Effect of different housing systems on productivity and welfare of laying hens	Review
Sparks	2008	Socio-economic drivers for UK organic pullet rearers and the implications for poultry health	Organic only

Sterling	2003	Relationships Among Strain, Performance, and Environmental Temperature in Commercial Laying Hens	Data from before 2000
Steward	2006	Assessment of Laying Hens Maintained in Different Housing Systems	Non-commercial
Stojčić	2012	Effect of genotype and housing system on egg production, egg quality and welfare of laying hens	Non-commercial
Stokholm	2010	Causes of Mortality in Commercial Organic Layers in Denmark	Organic only
Stratmann	2015	Soft perches in an aviary system reduce incidence of keel bone damage in laying hens	Cumulative mortality data (by age) not clear. Not clear how long is a laying period (mortality provided by laying period). Active culling of birds with lesions and locomotion problems.
Tactacan	2009	Performance and welfare of laying hens in conventional and enriched cages	Non-commercial
Tauson	2005	Mortality, production and use of facilities in furnished small group cages for layers in commercial egg production in Sweden 1998–2003.Proceedings of the 7:th European Symposium on Poultry Welfare, Lublin, Poland, June 2005. AnimalScience Papers and Reports.23: 95-102	Data from before 2000
Tavares	2018	Mortality, production and quality of eggs of different rearing systems	Mortality only over 30 days
Van	2009	A review of key health-related welfare issues in organic poultry production	Organic only
van der Meulen	2007	Questionnaire survey of disease prevalence and veterinary treatments in organic layer husbandry in the Netherlands	Organic only
Voslářová	2006	Comparison between laying hen performance in the cage system and the deep litter system on a diet free from animal protein	Non-commercial
Wang	2015	Investigating the welfare of laying hens in the conventional cage and floor production systems by Welfare Quality (CHINA)	Non-commercial
Weber	2003	Investigations of laying hen health in enriched cages as compared to conventional cages and a floor pen system	Non-commercial
Weeks	2007	Laywel: welfare implications of changes in production systems for laying hens. Deliverable 7.1 Overall strengths and weaknesses of each defined housing system for laying hens, and detailing the overall welfare impact of each housing system	Duplicate data with Blokhuis 2007
Weimer	2019	Laying hen production and welfare in enriched colony cages at different stocking densities	Non-commercial
Weitzenbürger	2005	Effect of furnished small group housing systems and furnished cages on mortality and causes of death in two layer strains	Non-commercial
Weżyk	2006	Quality traits of eggs from Hyline White and Hyline Brown hens kept in cages and on litter.	Non-commercial
Widowski	2017	Effect of space allowance and cage size on laying hens housed in furnished cages, Part I: Performance and well-being	Non-commercial
Yakubu	2007	Effects of Genotype and Housing System on the Laying Performance of Chickens in Different Seasons in the Semi-Humid Tropics	Non-commercial
Zuleima	2019	Caracterización Productiva del Período de Postura en Gallinas Lohman	Small sample (6000), no information on hen age

Table S2. Description of Sources included in the meta-analyses.

Data sources with information on cumulative laying hen mortality in commercially managed systems (2000-2020). CC: conventional cages; FC: furnished cages; ST: single-tier aviary; MT: multi-tier aviary; AV: aviary (type unspecified). All data and links to sources are available at https://osf.io/r5f6c.

Code	Source	Bibliographic details	Housing	Flocks	Country
AGR_14	Agrovision (2014)	Laying hen performance in different production systems; why do they differ and how to close the gap? European Poultry Science 78 (2014)	AV<br>FC	589<br>95	NLD
CAM_15	Campe et al (2015)	Determinants of economic success in egg production in Germany – here: laying hens kept in aviaries or small-group housing systems. Applied Agricultural and Forestry Research 65, 227–238 (2015).	MT<br>FC	47<br>18	DEU
ELS_06	i. Elson & Croxall (2006); ii. Elson (2015)	i. European study on the comparative welfare of laying hens in cage and non-cage systems. Archiv Fur Geflugelkunde 70, 194–198 (2006); ii. Poultry welfare in intensive and extensive production systems. Worlds. Poult. Sci. J. 71, 449–460 (2015).	CC<br>FC<br>MT<br>ST	4<br>3<br>4<br>6	GBR;DEU; NLD
FER_09	Ferrante et al (2009)	Effects of two different rearing systems (organic and barn) on production performance, animal welfare traits and egg quality characteristics in laying hens. Ital. J. Anim. Sci. 8, 165–174 (2009).	ST	1	ITA
FUL_19*	Fulton (2019)	Health of commercial egg laying chickens in different housing systems. Avian Dis. 63: 420-426 (2019)	CC<br>MT<br>FC	2<br>2<br>2	USA
GER_12	Gerzilov et al (2012)	Effect of poultry housing systems on egg production. Bulgarian Journal of Agricultural Science 18, 953–957 (2012).	CC<br>FC<br>ST	5<br>2<br>4	BGR
HEE_15	Heerkens et al (2015)	Specific characteristics of the aviary housing system affect plumage condition, mortality and production in laying hens. Poult. Sci. 94, 2008–2017 (2015).	MT	47	BEL
HOR_10	Horne et al (2010)	The poultry and pig sector in Argentina. Husbandry practice and animal welfare. (2010).	CC	210	ARG
WOR_13	Rubinoff (2013)	Rubinoff, I. Key global health issues in cage-free and organic laying hens (Hy-Line World Data on Mortality). in 5th International Veterinary Poultry Congress (Hy-Line International, 2016).	CC<br>AV	249<br>122	WOR
FRA_16	ITAVI (2016)	Performances techniques et coûts de production en volailles de chair, poulette et poules pondeuses. Resultat 2016	CC<br>ST<br>FC	1020<br>51<br>1020	FRA
LON_16	Long et al (2016)	Effect of light-emitting diode vs. fluorescent lighting on laying hens in aviary hen houses: Part 1 - Operational characteristics of lights and production traits of hens. Poult. Sci. 95, 1–11 (2016).	MT	4	USA
URY_16	MGAP Uruguay (2016)	Encuesta Postura Comercial 2014 - Oficina de Estadísticas Agropecuarias (DIEA)	CC	48	URY
NOR_18	Nortura (2018)	Nortura Eggkontroll Program, Data from Producers, Norway (2017-2019)	MT<br>FC	199<br>44	NOR
PET_15	Petrik et al (2015)	On-farm comparison of keel fracture prevalence and other welfare indicators in conventional cage and floor-housed	CC	9	CAN

		laying hens in Ontario, Canada. Poult. Sci. 94, 579–585 (2015).	ST	8	
RIB_17	Riber & Hinrichsen (2017)	Welfare Consequences of Omitting Beak Trimming in Barn Layers. Front Vet Sci 4, 222 (2017).	AV<br>AV	10<br>10	DEN
ROD_08	Rodenburg et al (2008)	Welfare assessment of laying hens in furnished cages and non-cage systems: assimilating expert opinion. Anim. Welf. 17, 355–361 (2008)	FC<br>ST<br>MT	6<br>4<br>3	BEL; DEU<br>NLD; BEL<br>NLD; BEL
SHI_19	Shini et al (2019)	Fatty liver haemorrhagic syndrome occurrence in laying hens: impact of production system. Avian Pathol. 48, 25–34 (2019).	CC	3	AUS
STA_16	Stadig et al (2016)	Survey of egg farmers regarding the ban on conventional cages in the EU and their opinion of alternative layer housing systems in Flanders, Belgium. Poult. Sci. 95, 715–725 (2016).	FC<br>ST<br>MT	19<br>44<br>53	BEL
TAH_14	Tahamtani et al (2014)	Does rearing laying hens in aviaries adversely affect long-term welfare following transfer to furnished cages? PLoS ONE 9: e107357 (2014).	FC	2	NOR
USA_13	USDA (2013)	Layers 2013 Part I: Reference of Health and Management Practices on Table-Egg Farms in the United States (2014).	CC<br>FC<br>AV	1074<br>53<br>171	USA
VST_18	van Staaveren et al (2018)	A description of laying hen husbandry and management practices in Canada. Animals 8, (2018).	FC<br>ST<br>MT	26<br>17<br>22	CAN
WEL_12	Weeks et al (2016): data from AssureWel(R) (2012)	Implications for welfare, productivity and sustainability of the variation in reported levels of mortality for laying hen flocks kept in different housing systems. Dataset: https://doi.org/10.5061/dryad.60q44	ST	9	GBR
FSA_09	Weeks et al (2016): data from FSA (2009)	"	CC<br>FC<br>ST	427<br>17<br>62	GBR
NIC_06	Weeks et al (2016): data from Nicol et al (2006)	"	ST	36	GBR
RIC_12	Weeks et al (2016): data from Richards et al (2012)	"	FC	11	GBR
SHE_10	Weeks et al (2016): data from Sherwin et al (2010)	"	CC<br>FC<br>ST	5<br>6<br>7	GBR
UFA_06	Weeks et al (2016): data from UFAW (2006)	"	FC<br>ST	6<br>10	GBR
WQP_12	Weeks et al (2016): data from Welfare Quality(R)	"	MT<br>CC<br>FC<br>ST	31<br>10<br>11<br>31	SWE; NLD

ZLO_18	Zloch et al (2018)	Influence of alternative husbandry systems on postmortem findings and prevalence of important bacteria and parasites in layers monitored from end of rearing until slaughter. Vet.Rec182:350 (2018)	AV	29	AUT

* FUL_19: same dataset as Karcher et al 2015.Poultry Science 94: 485-501

Table S3. Search Strategy for Web of Science (core collection) database
(Indexes=SCI-EXPANDED, SSCI, A&HCI, CPCI-S, CPCI-SSH, BKCI-S, BKCI-SSH, ESCI
Timespan=2000-2020; any document type)

No.	Search Strategy	Items found
#1.	TOPIC: ("laying hen" OR layer)	1,490,441
#2.	TOPIC: (aviar* OR cage* OR housing OR barn)	303,269
#3.	TOPIC: (mortality OR death OR performance OR survival OR productivity OR welfare)	6,099,867
#4.	#1 AND #2 AND #3	2,886

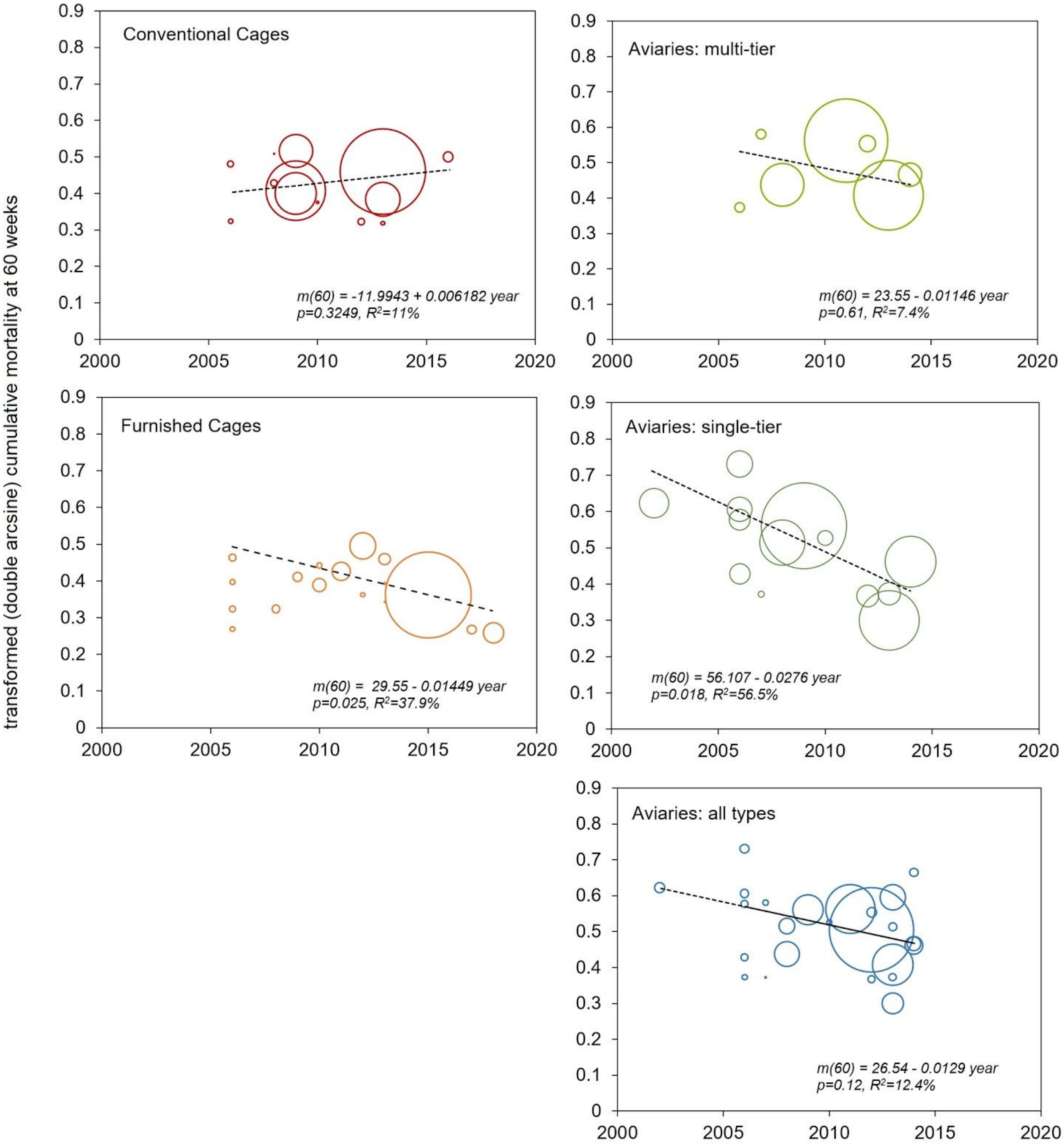

Figure S1. Meta-regression of cumulative laying hen mortality (m60, double arcsine transformed) in different indoor housing systems as a function of mid-year of data collection. Data sources for which we could not confirm that all flocks were kept exclusively indoors (multi-tier: HEE_15, NOR_18, VST_18; single-tier: VST_18; undefined aviary: USA_13) were excluded. Regression equations and statistical results are shown in each corresponding graph. Each circle corresponds to one data source, with circle sizes representing the weights from the meta-analysis.
